# Psoriasis Treatment Success With Bimekizumab: Two Hard-to-Treat Cases

**DOI:** 10.7759/cureus.114395

**Published:** 2026-08-12

**Authors:** Ahmed Ameen

**Affiliations:** 1 Dermatology, NMC Specialty Hospital, Abu Dhabi, ARE

**Keywords:** bimekizumab, case report, hard-to-treat patients, monoclonal antibody, psoriasis

## Abstract

Moderate-to-severe psoriasis can be a challenging disease for patients who are or become refractory to treatment, who are not eligible for some of the systemic drugs approved for psoriasis management, or who present with psoriasis in hard-to-treat areas. This is a case report that describes the effectiveness of bimekizumab in two hard-to-treat cases with moderate-to-severe psoriasis in the United Arab Emirates. Case 1 was a 32-year-old Filipino woman with psoriasis vulgaris manifesting on her legs and scalp after 15 years of failed topical, non-biologic oral systemic and biologic treatment. After one dose of bimekizumab, her lesions, disease activity, and quality of life scores had considerably improved. Case 2 was a middle-aged Emirati man with a five-year-long history of undiagnosed isolated nail psoriasis. After the initiation dose of bimekizumab, his nail psoriatic lesions had significantly improved, along with disease activity and quality of life scores. Both Case 1 and Case 2 patients had sustained clinical response seven months into their bimekizumab treatment, with no reported safety events. Bimekizumab can be considered the treatment of choice for patients who did not respond to other systemic treatments, as well as a first-line therapy for hard-to-treat moderate-to-severe psoriasis.

## Introduction

The prevalence of psoriasis is increasing worldwide [1]. The crude lifetime prevalence of psoriasis in the Arab world (North Africa and the Middle East) is estimated to range from 1.0 to 1.5% and from 0.5 to 1.0% in Southeast Asian countries [2]. Although psoriasis is less common in these two regions of the world compared to North America, Europe, and Australasia, literature on the epidemiology of psoriasis is limited, and the prevalence figures might therefore be inaccurate [2,3]. Nevertheless, psoriasis is associated with comorbidities, such as cardiovascular disease, mental health troubles, type 2 diabetes mellitus, stroke, Hodgkin and non-Hodgkin lymphoma, non-melanoma skin cancer, and inflammatory bowel disease (IBD) [1].

Particularly challenging and difficult-to-treat areas, such as nail, palmoplantar, or scalp lesions, are also prone to recurrence and to the appearance of new lesions, contributing to the physical, psychological, and economic burden of psoriasis [4-7]. Scalp psoriasis, which affects up to 80% of patients with psoriasis, is also particularly challenging given the difficulty of treatment delivery [8,9] and the abundant dandruff and itch that cause considerable psychosocial morbidity [8]. Fingernail psoriasis, being more visible than toenail psoriasis, is all the more distressing [10]. Concurrent nail psoriasis affects more than 50% of patients with plaque psoriasis [11], while isolated nail psoriasis has been reported in 5-10% of patients [12,13].

Therapeutic options for psoriasis include topical and systemic agents, with varying degrees of efficacy and various adverse effects. Scalp lesions can be topically treated with steroids, keratolytics, tar and anthralin compounds, vitamin D analogues, and vitamin A derivatives [8]. However, the effectiveness of these topical therapies remains limited, and better symptom clearance is achieved with agents such as methotrexate, cyclosporine, and oral retinoids [8] or approved biologics [9]. As recently reviewed, nail psoriasis is associated with a higher risk of joint involvement and arthritis, and topical treatments virtually offer no clinical benefits, motivating the use of biologics [10]. While interleukin 17 (IL-17) inhibitors (e.g, secukinumab) outperformed the previous biologics used for psoriasis treatment, including tumor necrosis factor alpha (TNF-α) blockers (e.g., adalimumab) and IL-12-23 inhibitors (e.g., ustekinumab), shifting from broad inflammatory cytokine inhibition to a more targeted approach, a dual IL-17A/IL-17F inhibition represents further refinement targeting key cytokines involved in psoriatic inflammation.

Bimekizumab is a first-in-class humanized monoclonal immunoglobulin G1 antibody that selectively inhibits both IL-17A and IL-17F, which are upregulated in psoriasis, as well as in other diseases with similar aetiology, such as psoriatic arthritis [14], hidradenitis suppurativa [15], and axial spondyloarthritis [16]. In patients with psoriasis, bimekizumab has proven superior to placebo (BE READY trial [17]), adalimumab (BE SURE trial [18]), ustekinumab (BE VIVID trial [19]), and secukinumab (BE RADIANT trial [20]) [21,22]. These trials culminated in the approval of bimekizumab for the treatment of moderate-to-severe psoriasis by the Food and Drug Administration (FDA) in 2023, following approval by the European Commission in 2021. In 2023, the European Centre for Guidelines Development (EuroGuiDerm), founded by the European Dermatology Forum (EDF), recommended bimekizumab as one of the preferred drugs for psoriasis patients who also suffer from advanced heart failure, ischemic heart disease, and concomitant latent/treated tuberculosis [23]. Of particular relevance, bimekizumab achieved considerable improvement in nail psoriasis manifestations, a widely acknowledged, difficult-to-treat type of psoriasis [20]. Guidelines for the treatment of moderate-to-severe psoriasis in the United Arab Emirates (UAE) were published in 2021 [24], i.e., before bimekizumab was granted European Commission or FDA approval. While this specific IL-17 inhibitor is not listed in the aforementioned consensus statement, UAE experts agree that biologics, including IL-17 inhibitors, can be prescribed as first-line treatment on a case-by-case basis, depending on disease severity, comorbidities, and location of lesions [24]. The benefits of IL-17 inhibitor therapy were discussed, including achieving Psoriasis Assessment Severity Index (PASI) 75 and PASI 100 response by Week 12, while nasopharyngitis, headache, upper respiratory tract infection, and arthralgia were the most commonly reported adverse events [24]. Additionally, the decision to continue with a therapeutic strategy or to switch to another treatment modality is carefully evaluated according to PASI [25] and the Dermatology Life Quality Index (DLQI) [26], as outlined by a French group of experts and adopted in the UAE [24,27]. Briefly, when reaching PASI ≤ 3, it is recommended to continue with the treatment strategy, while PASI > 3 and DLQI ≥ 5 motivate switching to another treatment [24].

In this manuscript, we describe for the first time two difficult-to-treat cases of psoriasis from the UAE, successfully managed by bimekizumab. Both patients demonstrated a rapid response after a single dose of treatment, adding to the growing body of evidence endorsing bimekizumab as a potent dual IL-17 inhibitor with a satisfactory safety profile.

## Case presentation

Material and methods

Patients with difficult-to-treat psoriasis who were treated at NMC Specialty Hospital in Abu Dhabi, UAE, had their data described in this case report. Patients in this case report were treated with bimekizumab according to drug availability and accessibility. Data on patients' journey and bimekizumab effectiveness were collected.

Ethics approval

This manuscript is a case report and is exempt from Institutional Review Board approval.

Consent for publication

Both patients had provided written consent to having their anonymous data and photographs of their lesions published. None of the reported data includes any information that can compromise patient confidentiality.

Case description

Case 1

A 32-year-old Filipino woman had a long history of psoriasis vulgaris for the past 15 years. The patient presented with erythematous thick plaques in both legs with scalp involvement, causing significant itching. She had a long journey of treatment for her chronic psoriasis, which started earlier in 2010 with systemic conventional therapy (methotrexate), proving ineffective. Her biological therapeutic journey started with secukinumab, the first IL-17 inhibitor, approved in 2015 [28]. After the 18-month favorable response started wavering, she was switched to ixekizumab (another IL-17A neutralizer) for nine months without satisfactory results, motivating treatment discontinuation. In 2020, she started risankizumab (IL-23A inhibitor) and responded to it for the first nine months, before completely losing clinical benefit by 14 months of treatment. At her first presentation to the clinic, she had been on guselkumab (IL-23 inhibitor) for seven months, but reported primary failure and treatment discontinuation (Table 1).

**Table 1:** Treatments prior to bimekizumab initiation

Case no.	Treatments prior to bimekizumab initiation	Duration of treatment (months)
Case 1	Methotrexate	-
	Secukinumab	18
	Ixekizumab	9
	Risankizumab	14
	Guselkumab	7
Case 2	Topical antifungal	12
	Oral antifungal	12

The patient was very depressed because of a long non-successful treatment journey with different biological therapies that failed to confer proper control of her disease. She reported being considerably affected by her disease progression and asked for urgent help. The patient's psoriasis severity was assessed according to the PASI, the Body Surface Area (BSA), and the DLQI (Table 2).

**Table 2:** Clinical benefits of bimekizumab treatment BSA: body surface area; DLQI: Dermatology Life Quality Index; NAPSI: Nail Psoriasis Severity Index; PASI: Psoriasis Area and Severity Index *Case 1 responded to bimekizumab treatment after 1 dose (i.e., 2 weeks of treatment), and Case 2 after the initiation dose (i.e., 16 weeks of treatment).

Assessment tool	Before bimekizumab treatment	At first treatment response*	After 7 months of treatment with bimekizumab
BSA	15%	4%	0%
DLQI	19/30	5/30	0/30
PASI	27.5/72	2/72	0/72
DLQI	22/30	15/30	8/30
NPASI	53/80	15/80	10/80

Based on her clinical assessment scores, the patient had moderate-to-severe psoriasis, which failed to be controlled with different treatment options, including multiple biological therapies.

Bimekizumab was then prescribed in October 2023, as per label posology, i.e., one dose of 320 mg (given as two subcutaneous injections of 160 mg each) every four weeks for a total of five initiation doses, followed by 320 mg (given as two subcutaneous injections of 160 mg each) every eight weeks (maintenance dose).

**Figure 1:**
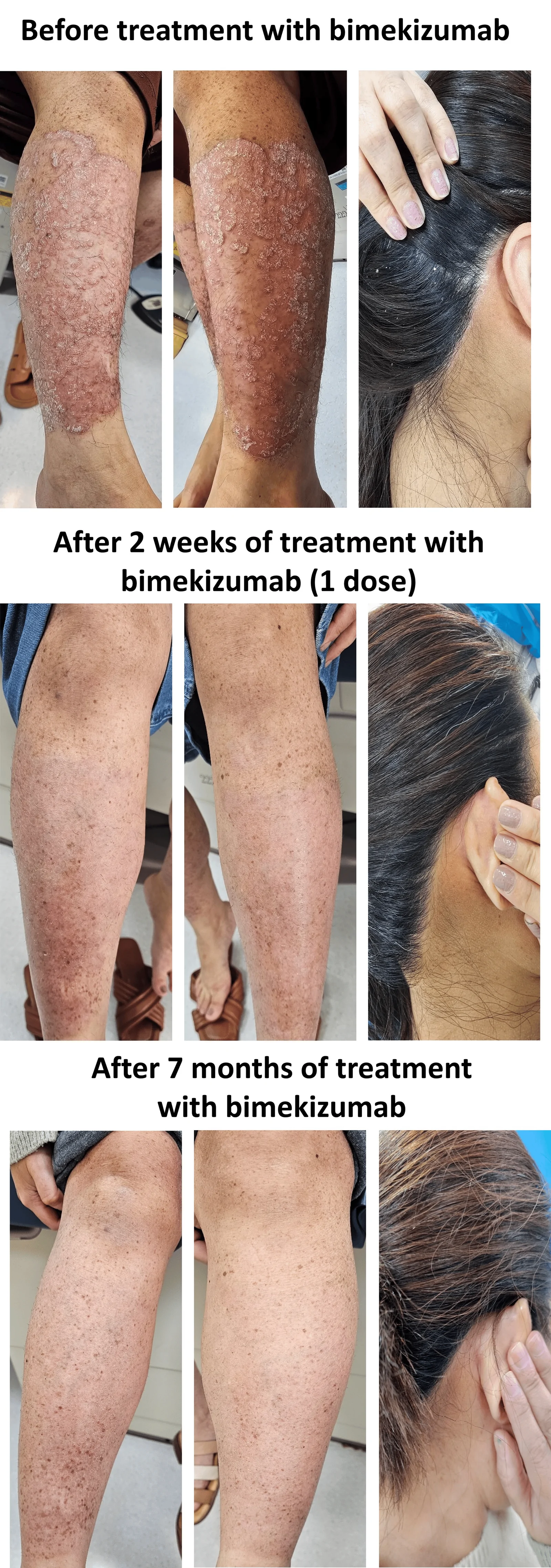
Evolution of psoriasis vulgaris over seven months of treatment with bimekizumab (Case 1)

Clinical improvement was visible after the first dose of bimekizumab, i.e., within two weeks of treatment initiation, with the patient reporting major skin and scalp clearance and complete control of itching. This favorable clinical response was maintained with subsequent doses (Table 2). The clinical improvement was documented by photographic evidence, displayed in Figure 1.

Case 2

A 55-year-old Emirati man had a five-year-long history of isolated nail psoriasis. The patient presented with nail pitting, subungual hyperkeratosis, and onycholysis, which was misdiagnosed earlier as onychomycosis. The patient was previously treated with topical and oral antifungal medication for one year, without any response (Table 1).

Clinical examination indicated that the patient met the diagnostic criteria of nail psoriasis rather than clinical or mycological evidence of onychomycosis. After careful skin and scalp examination, no other psoriatic lesions were identified, leading to the diagnosis of isolated nail psoriasis.

The patient had evident signs of anxiety and depression, due to his nail aspect, and he always wore gloves to hide his disease. He reported feeling embarassed of his condition and asked for desperate care for his long-standing problem.

The patient's nail psoriasis severity was assessed at the initial clinical examination by the Nail Psoriasis Assessment Severity Index (NPASI) and by the DLQI (Table 2).

**Figure 2:**
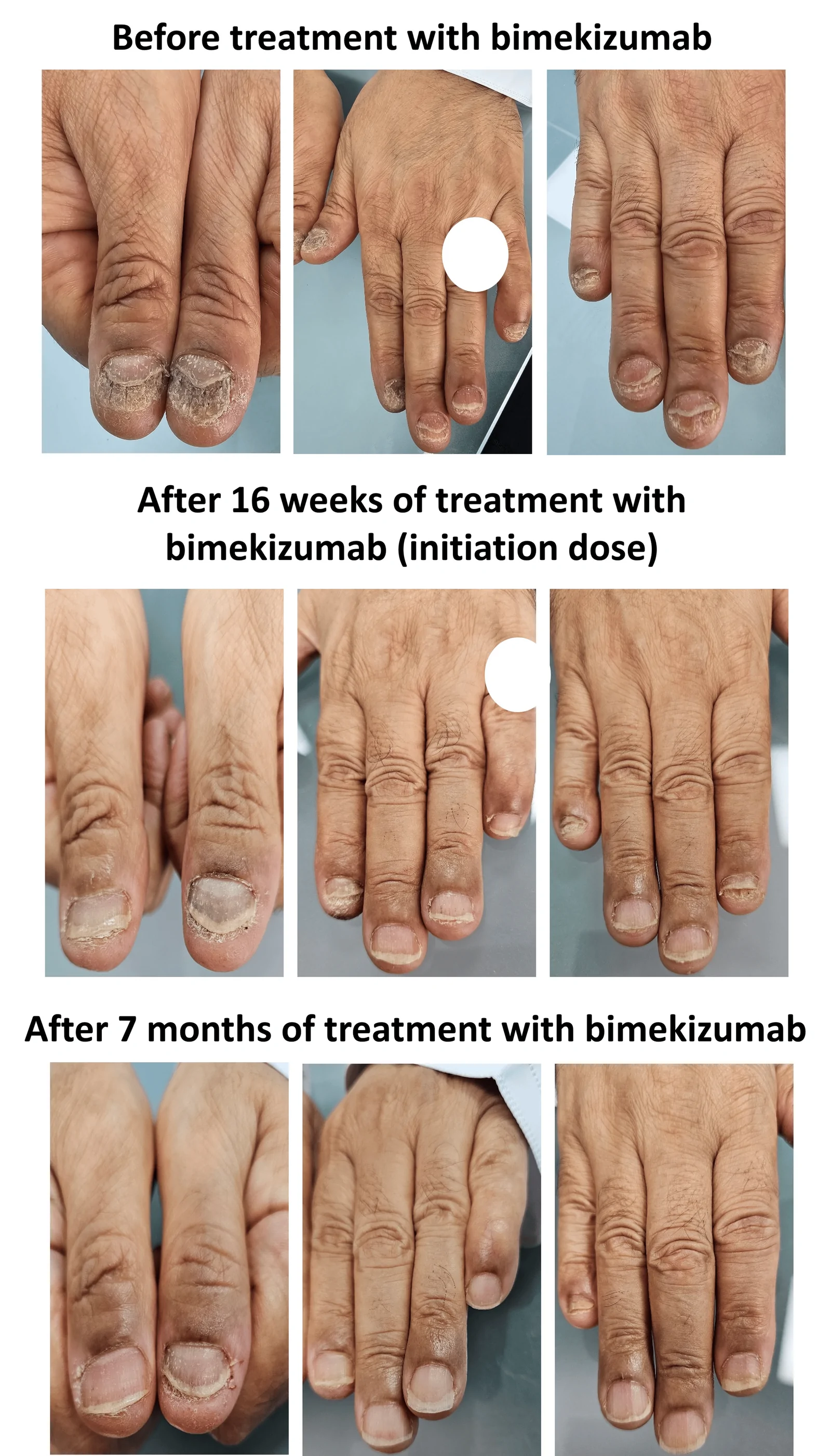
Evolution of nail psoriasis over seven months of treatment with bimekizumab (Case 2)

The patient had not previously received any psoriasis therapy, given a delayed diagnosis. He was started on bimekizumab in November 2023, as per label posology, i.e., one dose of 320 mg (given as two subcutaneous injections of 160 mg each) every four weeks for a total of five initiation doses, followed by 320 mg (given as two subcutaneous injections of 160 mg each) every eight weeks (maintenance dose). The patient reported considerable improvement after the initiation dose of bimekizumab, i.e., within 16 weeks of treatment initiation, and the response was maintained (Table 2). The clinical improvement was documented by photographic evidence, displayed in Figure 2.

## Discussion

The two cases reported above describe difficult-to-treat, yet quite common, manifestations of psoriasis. Treating patients presenting with refractory plaque psoriasis, including scalp involvement, as well as isolated nail psoriasis, are challenging situation. An effective treatment that will resolve clinical symptoms and alleviate the psychosocial burden is limited to therapies that are systemic (capable of reaching hard-to-treat areas), safe, and available in the country and healthcare facilities. The American Academy of Dermatology (AAD) 2019 guidelines reviewed IL-17 inhibitors that are approved for psoriasis treatment and recommended them for moderate-to-severe psoriasis, involving the scalp, nails, and other body areas [29]. In line with international practice [30], the UAE consensus endorses the use of biologics, including IL-17 inhibitors, as first-line treatment for some patients [24]. However, the AAD guidelines, as well as the local consensus on the management of psoriasis in the UAE, were both published before bimekizumab, a dual anti-IL-17 antibody, was approved for moderate-to-severe psoriasis [24]. Dual IL-17A/IL-17F inhibition provides broader suppression of the IL-17 pathway compared with selective IL-17A blockade (e.g., secukinumab and ixekizumab), enhancing the suppression of keratinocyte-driven inflammation, reducing inflammatory amplification, and potentially contributing to enhanced clearance in difficult-to-treat areas, such as the scalp and nails [31-33]. Bimekizumab was specifically endorsed for the treatment of moderate-to-severe psoriasis by the European Centre for Guidelines Development (EuroGuiDerm) in 2023 [23].

Case 1 patient had suffered multiple treatment failures, which complicated the decision on therapeutic strategy. However, her poor PASI and DLQI scores, coupled to affected BSA of 15%, justified the prescription of bimekizumab after failure of other systemic treatments, including other biologics [24]. Seven months (approximately 35 weeks) after treatment initiation with bimekizumab, the patient in Case 1 had achieved complete remission, with BSA and PASI at 0. This treatment success is in accordance with literature on phase II trials with bimekizumab, where PASI 75, PASI 90, and PASI 100 were achieved by week 12 of treatment, and PASI 90 was maintained by week 60 [34-36]. Phase III trials also saw bimekizumab achieve PASI 90 in 91% of patients, a durable response over the 56-week placebo-controlled trial duration [17]. Another comparator-controlled phase III trial also showed superiority of bimekizumab in achieving PASI 100 in 67% of patients by week 48 of treatment [19]. Importantly and of relevance to patients with psoriasis, Case 1 patient reported a DLQI of 5 (down from 19) after one dose of bimekizumab and of 0 by seven months of treatment, similar to the literature [17].

Case 2 patient was treatment-naïve until prescribed bimekizumab following his nail psoriasis diagnosis. While therapy usually commences with topical modalities, which escalate to non-biologic systemic treatments, de novo treatment with a biologic has been approved by UAE experts in the management of psoriasis affecting difficult-to-treat areas [24]. Specifically, the UAE recommendations stipulate that biologics can be prescribed as first-line treatment to patients with "Affected high-impact sites (DLQI >10), including hands and feet, face and scalp, genital area, and significant nail disease (regardless of BSA or PASI score)" [24]. British guidelines indicate that bimekizumab can only be prescribed in case other non-biologic systemic treatments failed to achieve a response or if these are contraindicated, while acknowledging the similar or superior efficacy of bimekizumab compared to several other biologics [30,37]. In the United States, an expert consensus panel also reiterated the superiority of bimekizumab and endorsed its use in patients with challenging manifestations of psoriasis, provided they do not suffer from IBD [30], which was shown to exacerbate or manifest upon treatment with an IL-17 inhibitor [30,38,39]. Neither Case 1 nor Case 2 patients described here had a known history of IBD or developed any signs and symptoms of IBD, or any other adverse event in the seven-month period following the start of bimekizumab.

Interestingly, none of the cases reported oral candidiasis, a recognized adverse event associated with dual IL-17A/F inhibition [17]. Case 2 was treated with oral antifungal only prior to bimekizumab treatment.

This two-case report provides clinical evidence on the use of bimekizumab for the treatment of challenging cases of psoriasis, namely, one case, involving the scalp, that was refractory to multiple treatment strategies, and the other case of isolated nail psoriasis. Both patients reported considerable improvement in their clinical symptoms and quality of life, experiencing no adverse events.

The rapid clinical improvement (i.e., as early as after the first dose of bimekizumab) was consistent with outcomes observed in a recently published case series of patients with severe, refractory psoriasis treated in Denmark, who had experienced multiple prior treatment failures, including several biologic therapies [31].

Limitations

The case series nature of the presented work describes only two cases and requires additional cases and larger studies to be able to generalize the effectiveness of bimekizumab in hard-to-treat patients with psoriasis. Some baseline clinical data were unavailable, which may have limited the characterization of the study population and the interpretation of treatment response. Additionally, the short follow-up period further limits outcomes and may restrict the interpretation of the reported outcomes.

## Conclusions

Bimekizumab can be considered a highly effective option for patients who did not respond to other systemic treatments, as well as a first-line therapy for hard-to-treat moderate-to-severe psoriasis.
